# Nucleolar stress induces a senescence-like phenotype in smooth muscle cells and promotes development of vascular degeneration

**DOI:** 10.18632/aging.104094

**Published:** 2020-11-04

**Authors:** Wenjing Zhang, Wen Cheng, Rosanna Parlato, Xiaosun Guo, Xiaopei Cui, Chaochao Dai, Lei Xu, Jiankang Zhu, Min Zhu, Kun Luo, Wencheng Zhang, Bo Dong, Jianli Wang, Fan Jiang

**Affiliations:** 1 Department of Physiology and Pathophysiology, School of Basic Medicine, Cheeloo College of Medicine, Shandong University, Jinan, Shandong Province, China; 2 Key Laboratory of Cardiovascular Proteomics of Shandong Province, Qilu Hospital of Shandong University, Jinan, China; 3 Key Laboratory of Cardiovascular Remodeling and Function Research, Chinese Ministry of Education, Chinese National Health Commission and Chinese Academy of Medical Sciences, and The State and Shandong Province Joint Key Laboratory of Translational Cardiovascular Medicine, Department of Cardiology, Qilu Hospital of Shandong University, Jinan, China; 4 Institute of Applied Physiology, University of Ulm, Ulm, Germany; 5 Institute of Anatomy and Cell Biology, University of Heidelberg, Heidelberg, Germany; 6 Department of Vascular Surgery, Shandong Provincial Hospital Affiliated to Shandong First Medical University, Jinan, China; 7 Department of General Surgery, The First Affiliated Hospital of Shandong First Medical University, Jinan, China; 8 Department of Transplant Surgery, Qilu Hospital of Shandong University, Jinan, China; 9 Department of Vascular Surgery, Shandong Provincial Hospital Affiliated to Shandong University, Jinan, China; 10 Department of Cardiology, Shandong Provincial Hospital Affiliated to Shandong University, Jinan, Shandong Province, China; 11 Current address: Department of Obstetrics and Gynecology, Qilu Hospital of Shandong University, Jinan, China

**Keywords:** TIF-IA, RNA polymerase I, aortic aneurysm, senescence, nucleolar stress response

## Abstract

Senescence of smooth muscle cells (SMCs) has a crucial role in the pathogenesis of abdominal aortic aneurysm (AAA), a disease of vascular degeneration. Perturbation of cellular ribosomal DNA (rDNA) transcription triggers nucleolar stress response. Previously we demonstrated that induction of nucleolar stress in SMCs elicited cell cycle arrest via the ataxia-telangiectasia mutated (ATM)/ATM- and Rad3-related (ATR)-p53 axis. However, the specific roles of nucleolar stress in vascular degeneration remain unexplored. In the present study, we demonstrated for the first time that in both human and animal AAA tissues, there were non-coordinated changes in the expression of RNA polymerase I machinery components, including a downregulation of transcription initiation factor-IA (TIF-IA). Genetic deletion of TIF-IA in SMCs in mice (smTIF-IA^-/-^) caused spontaneous aneurysm-like lesions in the aorta. In vitro, induction of nucleolar stress triggered a non-canonical DNA damage response, leading to p53 phosphorylation and a senescence-like phenotype in SMCs. In human AAA tissues, there was increased nucleolar stress in medial cells, accompanied by localized DNA damage response within the nucleolar compartment. Our data suggest that perturbed rDNA transcription and induction of nucleolar stress contribute to the pathogenesis of AAA. Moreover, smTIF-IA^-/-^ mice may be a novel animal model for studying spontaneous AAA-like vascular degenerations.

## INTRODUCTION

Abdominal aortic aneurysms (AAAs) are pathological dilations of the aortic lumen caused by degeneration of the vessel wall, most of which remain silent until growing to a large size and/or rupture of the aneurysmal sac [1]. AAA rupture is associated with a high mortality of around 80% in patients [1]. The aneurysmal aortic wall is characterized by intense inflammation and gradual disappearance of elastic fibers and smooth muscle cells (SMCs) [1, 2]. Malfunctions of SMCs, which are the most abundant cell type in the arterial wall, may play a pivotal role in AAA formation [3–6]. Apoptosis of SMCs is increased in AAA, and the loss of mass of SMCs may weaken the mechanical strength of vessel wall and limit its capability of producing extracellular matrix [3, 4]. In addition, accelerated cellular senescence of SMCs may also contribute to the pathogenesis of AAA [7, 8]. Studies have shown that replenishment of SMCs decelerates progression of AAA, probably by facilitating tissue regeneration [5, 6].

Cell senescence is a sequela of chronic insults by various stress conditions, among which oxidative stress, genotoxic stress, and replicative stress are well established examples [9, 10]. The nucleolus is the site where ribosomal DNA (rDNA) transcription and ribosome biogenesis occur. Of interest, the nucleolus may also act as an important cellular stress sensor and a mediator of the response to stress conditions. Inhibiting rDNA transcription (mediated by RNA polymerase I or Pol I) in eukaryotic cells triggers a conserved cellular stress response known as nucleolar stress (also termed ribosomal stress) [11, 12]. Induction of nucleolar stress causes activation of the p53 pathway [11, 12] thereby promoting cell senescence [13, 14]. Our previous study has demonstrated that, at least in vitro, both vascular endothelial and smooth muscle cells show nucleolar stress responses to various stress conditions [15]. Moreover, we have shown that induction of nucleolar stress elicits cell cycle arrest in SMCs via the ataxia-telangiectasia mutated (ATM)/ATM- and Rad3-related (ATR)-p53 axis [16], and mitigates the development of proliferative vascular diseases [16, 17].

Nucleolar stress may be involved in the pathogenesis of neuronal degenerations [18–21]. However, potential implications of nucleolar stress in vascular degenerative diseases remain unexplored. To further understand the relationship between rDNA transcription/nucleolar stress and AAA development, we employed a genetic mouse model of conditional ablation of transcription initiation factor-IA (TIF-IA) in SMCs. TIF-IA (the homologue of RRN3 in yeast) is a critical component of the Pol I machinery, which mediates the association between an initiation-competent Pol I and the preinitiation complex, which is composed of upstream-binding factor (UBF) and SL-1 (a complex containing the TATA box-binding protein TBP) [22, 23]. Assembly of a complete Pol I machinery at the rDNA promoter region is the first step in the process of pre-rRNA synthesis. Although global knockout of TIF-IA results in embryonic lethality at day 9.5 [24], cell-specific knockout of TIF-IA by the Cre-loxP system has been proved to be a useful tool to study mechanisms of nucleolar stress associated pathologies [18–21].

## RESULTS

### AAA tissues show altered expression of various components of the Pol I machinery

**Figure 1:**
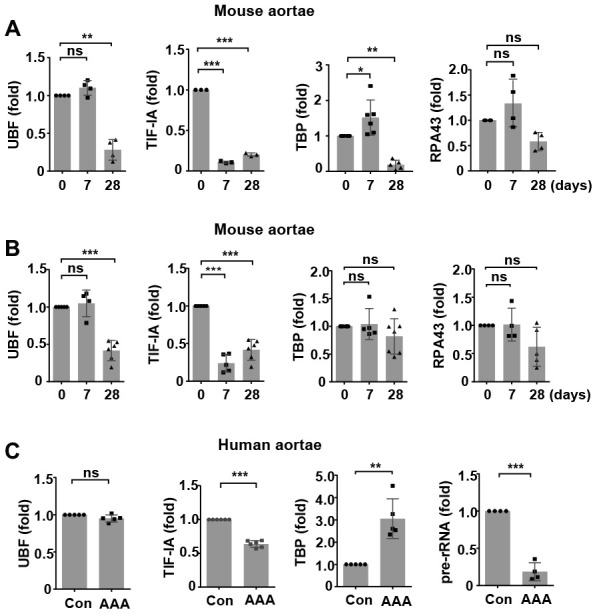
**Altered expressions of components of the RNA polymerase I (Pol I) machinery in animal and human abdominal aortic aneurysm (AAA) tissues.** (**A**) Real-time PCR results showing time-dependent changes of various Pol I components in wild type mouse abdominal aortas with CaCl_2_/phosphate-induced AAA (*n* = 3 - 6). (**B**) Real-time PCR results showing time-dependent changes of various Pol I components in apolipoprotein E (ApoE)^-/-^ mouse abdominal aortas with angiotensin II-induced AAA (*n* = 4 - 7). (**C**) Real-time PCR results showing changes in the expression of various Pol I components and pre-rRNA in human AAA tissues as compared to normal aortas (Con) (*n* = 4 - 6). The level of 45S pre-rRNA was normalized with the total 18S mature rRNA. For other genes, GAPDH or β-actin was used as the housekeeping gene. Dot blot-combined bar graphs represented mean ± S.D. * *P* < 0.05, ** *P* < 0.01, *** *P* < 0.001, unpaired *t*-test or one-way ANOVA as appropriate. The *n* number represents biological replications (same for all figures). ns, no significance.

We first characterized time-dependent changes in the expression of various components of the rDNA transcriptional machinery in two murine models of AAA. In the abdominal aorta from C57BL/6 mice with CaCl_2_/PBS-induced AAA, the expression levels of UBF, TBP and RPA43 (the TIF-IA interacting subunit of Pol I) [25] were not altered at day 7, while the level of TIF-IA was reduced (Figure 1A). At day 28, expressions of all of these factors were downregulated (Figure 1A). In the abdominal aorta from ApoE^-/-^ mice with angiotensin II infusion, the expression of UBF was not changed at day 7 but was decreased at day 28 (Figure 1B). The expression of TIF-IA was significantly downregulated at both day 7 and day 28 (Figure 1B). In comparison, the expressions of TBP or RPA43 were not significantly altered at either time points. Next, we examined whether there were changes in the expression of Pol I machinery components in human AAAs. As compared to normal aortas, human AAA tissues exhibited a downregulation in the expression of TIF-IA, whereas the level of TBP was upregulated (Figure 1C). The expression of UBF was not changed. To determine how the divergent changes of these Pol I components in human AAA affected rDNA transcription, we measured the ratio of 45S pre-rRNA to total 18S rRNA. As shown in Figure 1C, the relative amount of pre-rRNA was reduced in AAA, indicating the presence of perturbed rDNA transcription.

### Smooth muscle-specific TIF-IA deficiency causes spontaneous aneurysm-like lesions in the aorta

**Figure 2:**
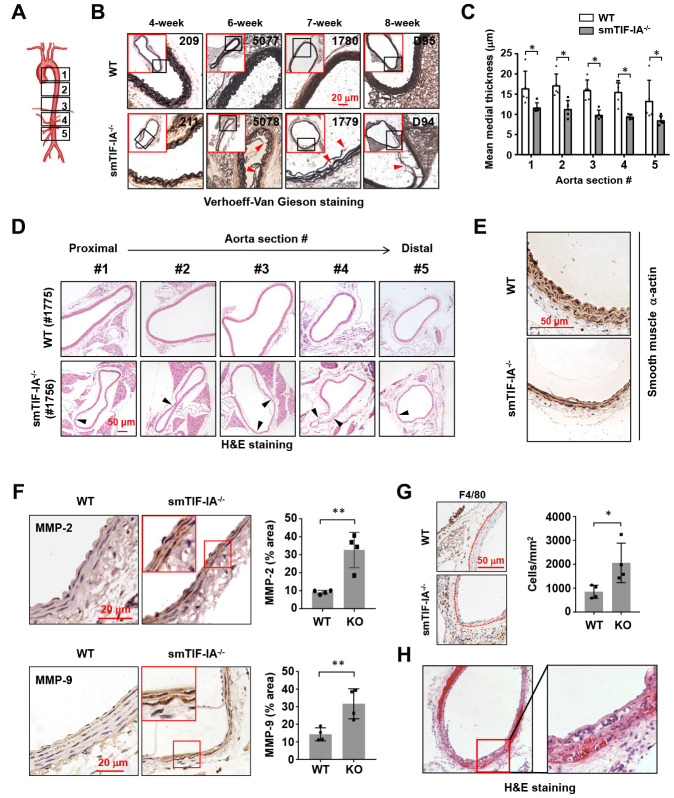
**Smooth muscle-specific nucleolar stress induction by TIF-IA deletion (smTIF-IA^-/-^) resulted in aneurysm-like lesions in the aorta.** (**A**) Depiction of sampling locations along the aorta for histopathological examinations. The aorta was divided into five parts from the proximal to the distal end. (**B**) Representative images of Verhoeff-Van Gieson staining of abdominal aortas (section #4 as shown in **A**) from sex-matched littermate wild type (WT) and smTIF-IA^-/-^ animals of different age. The red-outlined insets were low power images indicating the area where the corresponding high power images were taken. Sites with elastic laminar derangements were indicated by arrowheads. Animal identification tag numbers were shown at the top right corner. (**C**) Changes in the mean medial thickness of smTIF-IA^-/-^ aorta as compared to WT controls (*n* = 5). (**D**) H&E staining images showing multiple aneurysm-like lesions (arrowheads) throughout a single aorta from smTIF-IA^-/-^ animal. (**E**) Immunohistochemical staining for smooth muscle α-actin (brown color) showing diminished smooth muscle cells in smTIF-IA^-/-^ aorta. (**F**) Immunohistochemical staining (brown color) and semi-quantitative data showing the increased levels of matrix metalloproteinase (MMP)-2 and MMP-9 in smTIF-IA^-/-^ (KO) aorta (*n* = 4). High power images were shown in red boxes. (**G**) F4/80 staining and quantitative data showing increased accumulation of macrophages in smTIF-IA^-/-^ abdominal aorta (*n* = 4). Dashed lines indicated the media-adventitia border. (**H**) Low and high power images of the abdominal aorta from one smTIF-IA^-/-^ animal sacrificed alive at 9 weeks showing abundant intramural red blood cells (H&E staining), indicating the presence of aortic dissection. Quantitative data were expressed as dot blot-combined bar graphs representing mean ± S.D. * *P* < 0.05, ** *P* < 0.01, unpaired *t*-test.

To investigate the potential involvement of nucleolar stress in AAA pathogenesis, we analyzed the vascular phenotype of smTIF-IA^-/-^. These mice were viable at birth, but showed growth retardation, with the body weight being approximately 10-20% less than that of littermate wild type controls. Spontaneous mortalities were observed between 4 to 10 weeks (average 7.3 weeks, *n* = 7). The death was occurring without obvious preceding behavioral abnormalities. We performed autopsies for these mice, but did not find bleedings in the abdominal or thoracic cavity. Histopathological examinations revealed that 100% of smTIF-IA^-/-^ mice harbored wide spread aneurysm-like lesions in both of the thoracic and abdominal aortas. To confirm the above findings with autopsy, we sacrificed additional smTIF-IA^-/-^ mice alive at time points between 4 to 9 weeks. Similarly, aneurysm-like lesions were observed in all of the aortas (*n* = 7). The vascular lesions exhibited derangement and fragmentation of the elastic lamina, and diminished SMCs (Figure 2A–2E). The mean medial thickness of the vessel wall was decreased in the smTIF-IA^-/-^ aorta (Figure 2C). Some lesions in the abdominal aorta were associated with a moderate luminal expansion (Figure 2D); however, bulging dilatations typically found in human AAAs were not observed in these models. The aortic lesions were similarly observed in both male and female animals. To determine whether the deterioration of the aortic tissue in smTIF-IA^-/-^ mice was related to high blood pressure, we continuously monitored the conscious blood pressure for an 8-day period. We found that the blood pressure in smTIF-IA^-/-^ mice appeared to be slightly lower as compared to wild type controls (Supplementary Figure 1). Immunohistochemistry analysis demonstrated that smTIF-IA^-/-^ aortas exhibited increased levels of matrix metalloproteinase (MMP)-2 and MMP-9 (Figure 2F). Moreover, there was an increase in macrophage accumulation in the perivascular region of smTIF-IA^-/-^ aorta (Figure 2G), especially in the abdominal aorta but not in the thoracic aorta. Interestingly, in one smTIF-IA^-/-^ mouse sacrificed alive at 9 weeks of age, there were abundant intramural red blood cells in the abdominal aorta, indicating the occurrence of aortic dissection (Figure 2H).

### Inhibiting rDNA transcription accelerates AAA formation

**Figure 3:**
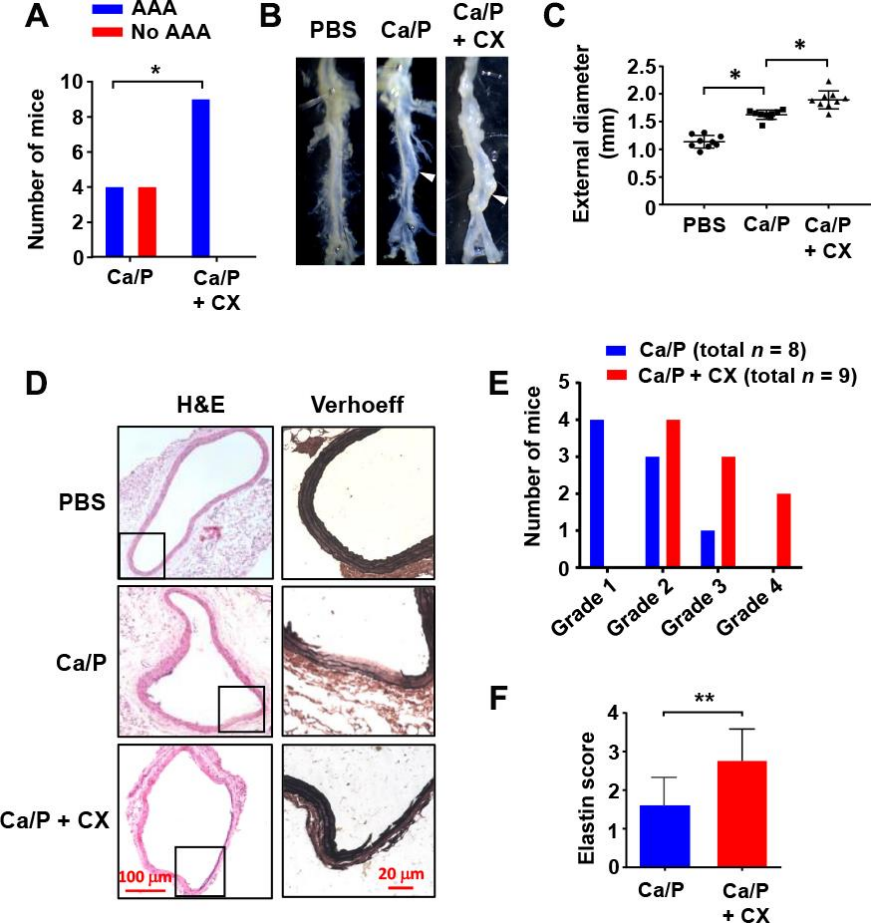
**Nucleolar stress induction accelerated AAA formation in vivo.** (**A**) Overall incidences of AAA in CaCl_2_/phosphate (Ca/P)-treated abdominal aortas in wild type C57BL/6 mice without and with CX-5461 (CX) co-treatment. (**B**) Gross morphology of Ca/P-induced AAAs (arrowheads) without and with CX-5461 co-treatment. PBS was used as sham control. (**C**) Dot blots showing the maximal diameter of the abdominal aorta in different groups (*n* = 8 - 9). (**D**) Histopathology of Ca/P-induced AAAs without and with CX-5461 co-treatment. The boxes within H&E-stained images indicated the areas shown on the right, which were Verhoeff-Van Gieson-stained high power images. (**E**) Proportions of AAAs with different severity (graded 1 to 4 with increasing severity) in Ca/P-treated aortas without and with CX-5461 co-treatment. (**F**) Elastin Scores of Ca/P-induced AAAs without and with CX-5461 co-treatment (*n* = 8 - 9). Data were expressed as mean ± S.D. Data in C and F were analyzed with one-way ANOVA and unpaired *t*-test respectively. Data in A were analyzed using χ^2^ test. * *P* < 0.05, ** *P* < 0.01.

To further clarify whether perturbed rDNA transcription had a pathogenic role in AAA formation, we utilized the selective Pol I inhibitor CX-5461. CX-5461 prevents the recruitment of SL-1 to the rDNA promoter, thereby blocking the subsequent tethering of Pol I, an effect analogous to that of TIF-IA deficiency [26]. We demonstrated that perivascular treatment with CX-5461 aggravated the development of AAA induced by CaCl_2_/phosphate in wild type mice. CX-5461 increased the incidence of AAA formation following CaCl_2_/phosphate treatment. The average diameter of CX-5461-treated abdominal aortas was significantly greater than that of controls (Figure 3A–3C). Histopathological analysis revealed that in CX-5461-treated aortas, elastic lamina fragmentation and loss of medial SMCs were more prominent (Figure 3D). We counted the incidence of different types of AAA according to their pathological classifications [27], and showed that the number of high-severity AAAs was increased in CX-5461-treated aortas (Figure 3E). Moreover, we assessed the severity of elastic lamina destruction using an Elastin Score as described [27]. It was shown that the Elastin Score was significantly higher in AAAs with CX-5461 treatment (Figure 3F).

### Nucleolar stress induces a senescence-like phenotype in vascular SMCs

**Figure 4:**
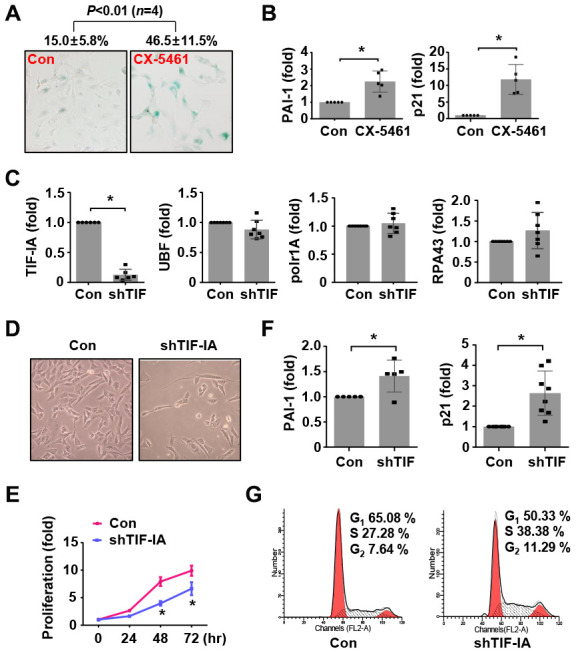
**Nucleolar stress caused a senescence-like phenotype in murine vascular SMCs (MOVAS).** (**A**) CX-5461 treatment (0.5 μM for 5 days) increased the number of β-galactosidase (β-Gal) positive cells (blue color). The numeric data were shown on top. (**B**) Real-time PCR results showing the expression levels of plasminogen activator inhibitor (PAI)-1 and p21^Cip1^ in control and CX-5461-treated (0.5 μM for 48 hr) cells (*n* = 5). (**C**) Real-time PCR results showing the effects of TIF-IA-targeting short hairpin RNA (shTIF) on the expressions of TIF-IA and unrelated Pol I components (*n* = 6 - 7). (**D**) Changed morphology of shTIF-IA-treated cells comparing to control cells. (**E**) Proliferation of control and shTIF-IA-treated cells assessed by CCK-8 assay (*n* = 3). (**F**) Changes in the mRNA levels of PAI-1 and p21^Cip1^ in shTIF-IA-treatment cells (*n* = 5 - 8). (**G**) Flow cytometry data showing G2/M blockade and S phase delay in shTIF-IA-treated cells. Data were expressed as mean ± S.D. * *P* < 0.05 versus control (Con), unpaired *t*-test. Flow cytometry assays were repeated at least 3 times.

Since there was evidence suggesting that the development of AAA was associated with SMC senescence, we hence examined whether perturbed rDNA transcription could affect SMC senescence. Previously we found that inhibition of Pol I with CX-5461 induced G2/M cell cycle arrest in SMCs [16]. Here we showed that CX-5461 treatment of MOVAS cells increased the number of senescent cells as revealed by β-Gal staining (Figure 4A), and this effect was accompanied by upregulations of the senescence markers plasminogen activator inhibitor (PAI)-1 and p21^Cip1^ (Figure 4B). Moreover, CX-5461 treatment upregulated another senescence marker p16^INK4a^, as well as markers of the senescence-associated secretory phenotype including IL-6 and IL-8 (Supplementary Figure 2). In order to exclude possible off-target effects of CX-5461, we treated MOVAS with another Pol I inhibitor BMH-21. BMH-21 showed similar stimulating effects on p21^Cip1^, p16^INK4a^, PAI-1, IL-6 and IL-8 expressions (Supplementary Figure 2).

Next, we tested whether TIF-IA knockdown had similar effects as CX-5461 by using lentiviral vectors expressing TIF-IA shRNA. Validation experiments demonstrated that TIF-IA shRNA transduction diminished the expression of TIF-IA, but did not affect other Pol I co-factors including UBF, RPA43 or Polr1A (Figure 4C and Supplementary Figure 3). SMCs with TIF-IA knockdown proliferated slower than control cells (Figure 4D and 4E). Consistently, TIF-IA knockdown also increased the levels of PAI-1 and p21^Cip1^ (Figure 4F). Similar to the effect of CX-5461 [16], TIF-IA knockdown increased the number of cells in G2 (Figure 4G), indicating the presence of a G2/M blockade. We also observed that the number of cells in S phase was also augmented in TIF-IA-silenced cells, indicating that TIF-IA deficiency induced a S phase delay as did the Pol I inhibitor in previous reports [28]. In addition, IL-6 and IL-8 were all upregulated in TIF-IA-silenced cells (Supplementary Figure 2).

To further corroborate the results from MOVAS cells, we treated human aortic smooth muscle cells with CX-5461 and/or BMH-21. It was demonstrated that BMH-21 increased the number of β-Gal-positive cells; both of BMH-21 and CX-5461 increased the expression levels of p21^Cip1^, p16^INK4a^ and PAI-1 (Supplementary Figure 4). Western blot experiments also confirmed the increased p21^Cip1^ expression in CX-5461-treated human smooth muscle cells (Supplementary Figure 4).

### Nucleolar stress elicits a DNA damage response in SMCs

**Figure 5:**
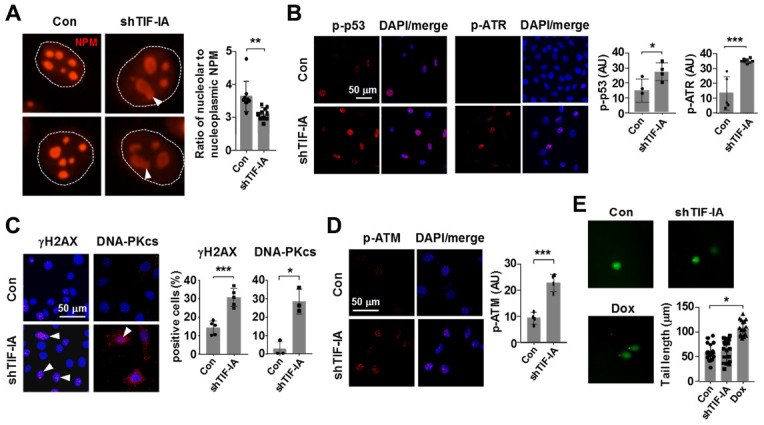
**Nucleolar stress elicited a DNA damage response in vascular SMCs (MOVAS).** (**A**) Immunofluorescence staining for nucleophosmin (NPM) showing that TIF-IA silencing triggered a nucleolar stress response as evidenced by the appearance of nucleolar caps (arrowheads) and redistribution of NPM from nucleoli to the nucleoplasmic space. Single nuclei were outlined by the dashed line. The graph on the right showed changes in the ratio of nucleolar to nucleoplasmic NPM fluorescence intensity (*n* = 9 - 10). (**B**) Immunofluorescence images and semi-quantitative mean intensity data expressed in arbitrary units (AU) showing the increased phosphorylations of p53 and ATR in TIF-IA-silenced cells (*n* = 3 - 8). Nuclei were counterstained with DAPI (blue). (**C**) Immunofluorescence images and quantitative data showing accumulation of γH2AX and DNA-PKcs foci (arrowheads) in the nuclei of TIF-IA-silenced cells (*n* = 3 - 5). (**D**) Immunofluorescence and the mean intensity data showing the increased phosphorylation of ATM in TIF-IA-silenced cells (*n* = 4). (**E**) Alkaline comet assay results showing that TIF-IA knockdown did not cause massive DNA breaks (*n* = 17 cells measured). Doxorubicin (Dox, 1 μM) was used as a positive control. Data were mean ± S.D. * *P* < 0.05, ** *P* < 0.01, *** *P* < 0.001 versus control (Con), unpaired *t*-test or one-way ANOVA as appropriate.

Previously we found that inhibiting Pol I in SMCs with CX-5461 produced a robust response of p53 phosphorylation, rather than increasing the total amount of p53 protein [16]. Evidence from us and others has also shown that inhibiting Pol I triggers a non-canonical DNA damage response featured by activation of the ATM/ATR pathway, which may explain the induction of p53 phosphorylation [16, 28–30]. However, some evidence also indicates that CX-5461 acts as a stabilizer of G-quadruplex DNA, which may potentially cause DNA damages independent of Pol I inhibition [31]. Hence, whether disrupting the normal Pol I machinery can directly elicit DNA damage responses is still an enigma. To address this question, we knocked down TIF-IA expression in SMCs and examined various markers of the DNA damage response. We first confirmed that TIF-IA gene silencing caused a moderate nucleolar stress response as evidenced by the reduced nucleolar compartmentalization of nucleophosmin (NPM) and emergence of nucleolar caps [32] (Figure 5A). In addition, we confirmed that TIF-IA-silenced cells exhibited increases in phosphorylation of p53 and ATR, effects similar to those induced by CX-5461 (Figure 5B and Supplementary Figure 5). Next we demonstrated that TIF-IA silencing increased the proportion of cells with γH2AX and DNA-PKcs foci, indicating the presence of DNA double strand breaks (DSBs) (Figure 5C). Moreover, TIF-IA silencing increased the level of ATM phosphorylation, which was thought to be an effector response to DSBs (Figure 5D). However, alkaline comet assay experiments showed that there was not a significant increase in the tail length in TIF-IA-silenced cells (Figure 5E).

### Increases in cellular senescence, nucleolar stress, p53 phosphorylation and DNA damage response in aneurysmal aortic tissues

**Figure 6:**
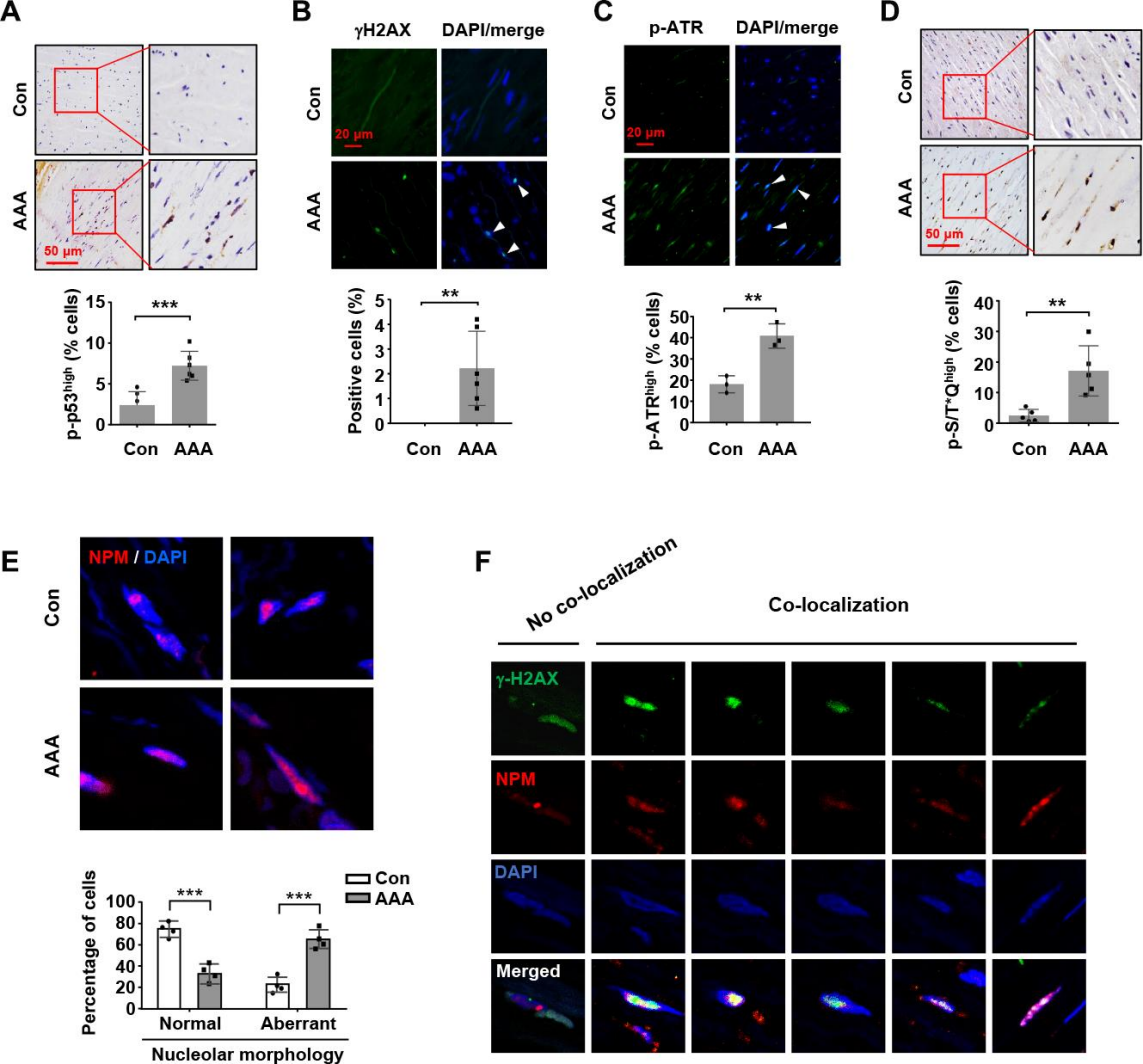
**Increases in nucleolar stress response, p53 phosphorylation and DNA damage response in human AAA tissues.** (**A**) Immunohistochemical staining and semi-quantitative data showing the increased level of p53 phosphorylation in the medial layer of normal (Con) and AAA aortas (*n* = 6). (**B**) Immunofluorescence results showing the increased number of γH2AX foci (arrowheads) in AAA tissues (*n* = 6). (**C**) Immunofluorescence results showing the increased number of cells exhibiting a high level of ATR phosphorylation (arrowheads) in AAA tissues (*n* = 3). (**D**) Immunohistochemical staining and semi-quantitative data showing the increased level of phospho-S/T*Q motif of ATM/ATR substrates in AAA tissues (*n* = 5). (**E**) Immunofluorescence images of NPM staining showing medial cells with normal nucleoli morphology (examples from control aortas) and those with aberrant nucleoli morphology (highly diffused NPM fluorescence signal) (examples from AAA tissues). The quantitative data below showed the proportions of cells with normal and aberrant nucleoli morphology in control and AAA aortas (*n* = 4). (**F**) Example images of AAA medial cells positive for γH2AX foci showing non-co-localization and co-localization of γH2AX with NPM. Dot blot-combined bar graphs represented mean ± S.D. ** *P* < 0.01, *** *P* < 0.001 versus control, unpaired *t*-test.

First, we confirmed that the expressions of PAI-1, p21^Cip1^, MMP-2 and MMP-9 were all upregulated in human AAA tissues (Supplementary Figure 6); these data were consistent with previous findings by other groups, indicating that an increase in cell senescence, especially in the medial smooth muscle cells, was a prominent characteristic of human aneurysmal vessel wall [7, 8]. Increased p53 accumulation has been observed in human AAA tissues [4]. However, currently there is no information about changes in the phosphorylation status of p53 in AAAs from either human or animal studies. This question is important because post-translational modifications (particularly phosphorylation on serine 15) have fundamental impacts on the transcriptional function of p53 [33]. To address this question, we performed immunohistochemistry and immunofluorescence analyses. As explained in Methods, for final data analyses in these experiments, we only included the smooth muscle-rich areas with minimal macrophage infiltrations, as pre-defined in preliminary experiments (see Supplementary Figure 7). We demonstrated that the level of p53 phosphorylation (Ser15) was significantly increased in the medial layer of AAAs (Figure 6A). In addition, several studies have revealed that aneurysmal SMCs accumulate DNA damages over time [34, 35]. To corroborate these results, we performed γH2AX staining in aortic sections, and demonstrated that the prevalence of cells exhibiting positive γH2AX staining in the nuclei was significantly increased in AAA tissues (Figure 6B). In comparison to the ATM-γH2AX axis, activation of ATR is thought to be primarily driven by exposure of single strand DNA. Hence we also measured ATR phosphorylation with immuno-staining, which demonstrated that the level of phospho-ATR was also increased in the medial layer of AAA (Figure 6C). Moreover, the phosphorylation level of the ATM/ATR substrate S/T*Q motif was augmented in AAA tissues (Figure 6D). To clarify whether AAA was associated with increased nucleolar stress, we performed immunofluorescence staining for NPM. It was found that the number of cells with aberrant nucleoli morphology (exhibiting diffused NPM fluorescence signal) was increased in AAA tissues (Figure 6E).

Despite the above findings, however, it is still unknown whether there is a connection between the increased DNA damage response in AAA tissues with perturbations of the rDNA genome. To explore this possibility, we carefully analyzed the medial cells in AAA samples by performing double immunofluorescence labeling for γH2AX and NPM. We surveyed cells with positive staining of γH2AX foci, and classified these cells according to the spatial relationship of γH2AX and NPM. Interestingly, we found that over half of the γH2AX-positive cells showed co-localization of the γH2AX and NPM signals (Figure 6F). On the other hand, most of the γH2AX-positive cells showing no γH2AX-NPM co-localization had intact nucleoli (as indicated by the relatively confined NMP fluorescence signal) (Figure 6F). These data suggest that in AAA tissues, occurrence of nucleolar stress response is associated with an increased incidence of DNA damages within the nucleolar compartment (see Discussion below). Using double fluorescent labeling, we further confirmed that the majority of γH2AX signals co-localized with smooth muscle α-actin (Supplementary Figure 8).

To further confirm our findings in the smTIF-IA^-/-^ model, we performed immunohistochemical staining, and demonstrated that phospho-p53 and phospho-ATR levels were all elevated in the tunica media of TIF-IA^-/-^ aorta (Supplementary Figure 9). β-Gal staining confirmed that the number of senescent cells was also increased in smTIF-IA^-/-^ aortas (Supplementary Figure 9). However, we found that there was not a significant difference in the number of apoptotic cells in the media between wild type and smTIF-IA^-/-^ models (Supplementary Figure 9). Furthermore, smTIF-IA^-/-^ aortas showed increased levels of IL-8 and PAI-1 in the tunica media as compared to wild type controls (Supplementary Figure 10).

## DISCUSSION

In the present study we investigated the impact of nucleolar stress on the pathogenesis of AAA. We showed that both human and animal models of AAA are associated with aberrant expression of components of the Pol I machinery. Specifically, the Pol I co-factor TIF-IA is consistently downregulated in all of the animal and human AAA tissues, whereas changes of other Pol I components appear to be variable depending on the etiology and/or time course of the disease. The non-coordinated changes in Pol I machinery expression are accompanied by enhanced nucleolar stress response and decreased pre-rRNA production in AAA tissues, suggesting that the basal homeostasis of rDNA transcription is perturbed in AAA. Furthermore, we have shown that nucleolar stress induction in SMCs causes spontaneous aneurysm-like lesions in the aorta in vivo. In vitro, nucleolar stress results in activation of the p53 pathway and a senescence-like phenotype in SMCs. These data together suggest that perturbed rDNA transcription and induction of nucleolar stress response in SMCs may contribute to the development of AAA.

TIF-IA is widely expressed in tissues and its expression is downregulated under cellular stress conditions such as amino acid withdrawal [23]. Our finding that TIF-IA expression is reduced in AAA tissues is supported by a previous genomic study, in which Biros and colleagues report that there is a 2-fold downregulation in TIF-IA gene expression in human AAA samples (adjusted *P* value equals 0.008) [36]. Although the mechanisms of the transcriptional regulation of TIF-IA in vascular SMCs are poorly understood, previous studies have suggested that the transcription of TIF-IA is under direct control of the oncogenic transcription factor c-Myc, which is a master regulator of rDNA transcription and is overexpressed in various cancers [23, 37]. In Myc-driven lymphoma, TIF-IA expression appears to be essential for maintaining cell survival [37]. In fact, c-Myc expression also has important roles in modulating vascular SMC functions, such as promoting cell proliferation [38]. Moreover, evidence suggests that c-Myc may act as a negative regulator of replicative senescence in vascular SMCs [39]. Nevertheless, it remains to be elucidated whether c-Myc is responsible for the decreased TIF-IA expression in AAA tissues.

Our previous study has shown that nucleolar stress does not induce massive apoptosis in primary SMCs in comparison to proliferating cancerous cells [16]. Similar results were also observed in post-mitotic neurons [18–21]. In the present experiments, we have observed that nucleolar stress induction by either TIF-IA knockdown or by CX-5461 treatment similarly induces a senescence-like phenotype in vascular SMCs, and this effect can be well explained by the activation of the DNA damage response-p53 axis. Currently, the nature of nucleolar stress-induced DNA damage response is elusive, and the experimental evidence is controversial about whether Pol I inhibition can actually cause DNA damages in cells [28, 40]. Our results with γH2AX and DNA-PKcs labeling, together with the increased level of ATM phosphorylation, suggest that occurrence of DSBs following Pol I inhibition cannot be ruled out, although the alkaline comet assay (detecting both of single and double strand breaks) has produced negative results. This may be due to the limited scope of DNA DSBs and/or a relatively efficient DNA repairing process in the cells [41, 42]. Importantly, the double labeling experiments in human AAA samples have demonstrated that around half of the γH2AX foci are localized in the nucleolar compartment. This evidence argues against that nucleolar DNA lesions are primarily caused by general genotoxic factors such as reactive oxygen species, which are expected to attack the genomic DNA in a random manner. Considering that the rDNA genome accounts for only ~0.4% of the whole genome [43], these data suggest a possibility of increased rDNA genome instability in AAA tissues [44, 45]. However, it should be noted that in human aneurysmal tissues, occurrence of nucleolar stress and its sequelae is unlikely to be smooth muscle specific; our data will not exclude the presence of nucleolar stress in other cell types.

We and others have shown that activation of the ATR arm of the DNA damage response may have a predominant functional role downstream of nucleolar stress induction in cells [16, 29]. Preferential induction of S-phase delay and G2/M blockade, but not significant activation of the G1 cell cycle checkpoint, are generally attributable to ATR activation [46]. Consistent with this notion, we have shown that nucleolar stress results in both G2/M arrest and S phase delay in vascular SMCs. Likewise, in human AAA samples we have observed a similar phenomenon that the prevalence of ATR activation appears to be greater than that of the γH2AX foci. How nucleolar stress activates ATR is still a mystery; nevertheless, this is unlikely to be due to massive unrepaired DNA single strand breaks, given the negative results from our alkaline comet assay. It is noted that, in contrast to ATM (which is primarily responsive to physical breakages of the DNA molecule), ATR is also readily activated by other types of genotoxic or non-genotoxic stimuli, such as stalled replication forks, hypoxia, and oxidative stress [46–48]. A mechanistic link between nucleolar stress and enhanced ATR activation in human aneurysmal SMCs warrants further investigations.

The aneurysmal lesion in the aorta caused by SMC-specific TIF-IA deletion reproduces key pathologic features of human AAA, including degeneration of the medial layer, diminished amount of SMCs, fragmentation of the elastic laminar, increased production of MMPs, and increased macrophage accumulation [2]. The vascular lesions are not caused by hypertension. In fact, the arterial blood pressure is moderately decreased in the smTIF-IA^-/-^ model, which could be related to impaired vasoconstriction functions of smTIF-IA^-/-^ smooth muscle cells. We argue that the fundamental pathogenic basis for all of these changes may be the senescence-like alterations of vascular SMCs. First of all, SMC senescence may compromise the injury repairing capability of the vessel wall [5, 6]. Secondly, senescent vascular SMCs may relapse into a state known as senescence-associated secretory phenotype, which is associated with increased MMP production, reduced collagen synthesis, and secretion of pro-inflammatory mediators [49]. Nevertheless, a limitation of the present study is that an intervention with senolytics is not included in the in vivo experiments to confirm a causal role of cellular senescence in the vascular pathology observed in smTIF-IA^-/-^ models. Hitherto, researchers have developed a number of animal models of AAA for pre-clinical studies [50–52]. However, most of the current AAA models require invasive surgical procedures and/or drug administration. Here we propose that the spontaneous aneurysm-like lesions in smTIF-IA^-/-^ mice render them a potential pre-clinical model for AAA studies. However, a limitation of the smTIF-IA^-/-^ model is the partial lethality that is unrelated to AAA. We suggest that an inducible smTIF-IA^-/-^ model might overcome this weakness.

In summary, we have shown that smooth muscle-specific disruption of the Pol I function causes spontaneous aneurysm-like degenerative lesions in the aorta, which are associated with nucleolar stress, activation of the ATM/ATR-p53 pathway, and induction of a senescence-like phenotype in SMCs. Together with the aberrant changes in Pol I machinery expression in human AAA tissues, our data strongly support the hypothesis that perturbations in rDNA transcription and induction of nucleolar stress in SMCs may contribute to the pathogenesis of vascular degenerative diseases such as AAA, although additional evidence is needed to show a direct link between nucleolar stress and smooth muscle senescence/degeneration in human aneurysmal vessels.

## MATERIALS AND METHODS

### Key reagents used in the study

Primary antibodies for NPM/B23 (ab10530), DNA-PKcs (ab70250), phospho-ATR (T1989) (ab227851), gamma γH2AX (S139) (ab26350), and phospho-ATM (S794) (ab119799) were obtained from Abcam (Cambridge, UK). Antibodies for phospho-p53 (Ser15) (#9284), phospho-ATM/ATR substrates (S/T*Q motif) (#9607), and MMP-2 (#40994) were from Cell Signaling (Beverley, MA, USA). Anti-MMP-9 was from R&D Systems (AF909) (Minneapolis, MN, USA). Anti-F4/80 was from Proteintech Group (28463-1-AP) (Wuhan, China). For short hairpin RNA (shRNA)-mediated gene silencing, 3 different constructs were designed and synthesized by Genepharma (Shanghai, China). The sequence showing the highest efficiency (5′-GCACAGACTGTCTTCCTTA -3′) was further cloned into pGLVU6/GFP lentiviral vector. Lentivirus packaging and purification were provided by Genepharma.

### Animal studies

All *in vivo* protocols were approved by the Institutional Animal Ethics Committee of Shandong University School of Medicine (Document LL-201501025), and performed in accordance with the Guide for the Care and Use of Laboratory Animals (NIH, USA). TIF-IA^flox/flox^ mice were previously generated [24]. To create smooth muscle-specific TIF-IA-deficient mice (smTIF-IA^-/-^), TIF-IA^flox/flox^ mice were crossed with the smooth muscle α-actin (SMA)-Cre transgenic strain, which was a gift from Professor Xiao Yang (State Key Laboratory of Proteomics, Collaborative Innovation Center for Cardiovascular Disorders, Genetic Laboratory of Development and Diseases, Institute of Biotechnology, Beijing, China) [53]. Since heterozygous TIF-IA^flox/+^SMA-Cre mice were normal and fertile, these mice were maintained as breeding pairs to generate smTIF-IA^-/-^. Littermate TIF-IA^+/+^ mice were used as wild type controls. The primers used for genotyping were listed in Supplementary Table 1. Male wild type C57BL/6 mice and apolipoprotein E-knockout (ApoE^-/-^) mice on C57BL/6 background were purchased from Vital River Laboratory (Beijing, China). Mice were housed in an air-conditioned environment with 12-hour light/dark cycles, and maintained on standard chow diet and water ad libitum.

CaCl_2_/phosphate-induced AAA was performed in C57BL/6 mice as previously described [54]. Briefly, mice of 10 to 12 weeks of age were anesthetized by isoflurane inhalation (5% for induction and 2% for maintenance). The infrarenal segment of the abdominal aorta was exposed and incubated with sterile 0.5 M CaCl_2_ solution for 10 min using a piece of soaked cotton slip. Subsequently the blood vessel was incubated with PBS for 5 min using the same method. The sham group received PBS incubation instead of CaCl_2_. Then the abdominal cavity was flushed with 0.9% NaCl. In order to inhibit Pol I, CX-5461 (purchased from Selleck Chemicals, Shanghai, China) was dissolved in DMSO and mixed in 25% cold Pluronic F-127 gel at 4^o^C to a final concentration of 1.0 μM. The gel solution of 50 μL was applied over the exposed abdominal aorta and allowed to solidify. The abdominal cavity was closed with a 4-0 suture, and the animal was allowed to recover on a warming pad and a single injection of analgesic (Carprofen 10 mg/kg subcutaneously) was given. Angiotensin II-induced AAA was performed in ApoE^−/−^ mice of 10 to 12 weeks of age [55]. Angiotensin II (from Abcam) was dissolved in sterile saline and loaded into osmotic mini-pumps (models 1007D or 2004 for different durations, Alzet, Cupertino, CA, USA). The pump was implanted under the skin on the back under isoflurane anesthesia (see above). Angiotensin II was delivered at a rate of 1000 ng/kg/min. Sham animals were infused with saline.

### Human tissue collection

Studies with human tissues were approved by the Human Ethics Committee of Shandong University Qilu Hospital (Document NO.2014-011), and conducted in accordance to the principles outlined in the Declaration of Helsinki. AAA specimens were obtained from patients who underwent AAA repair operations. Control aortic tissues were harvested from organ transplantation donors. Informed written consents were obtained from the patients or their direct relatives prior to the commencement of study. The background information of the patients was given in Supplementary Table 2.

### Histopathology and morphometry analyses

Mice were euthanized with Nembutal (Sigma-Aldrich, Saint Louis, MO, USA) at 100 mg/kg (intraperitoneal). For smTIF-IA^-/-^ models, the whole aorta excluding the ascending and arch areas was harvested and cut into five approximately equal parts. For induced AAA models, the abdominal aorta was cleaned and photographed under a stereo microscope (Model SZM-4, OPTIKA, Ponteranica, Italy) equipped with a CCD camera (SC600, OPTIKA). The maximal external diameter of the abdominal aorta was measured using the Image-Pro Plus software (Media Cybernetics, Bethesda, MD, USA). Mouse and human tissue blocks were fixed in 4% paraformaldehyde and embedded in paraffin. Serial cross sections of 4 μm thickness were cut and stained with the standard hematoxylin and eosin (H&E) staining or Verhoeff-Van Gieson staining (for visualization of elastic fibers). The slides were examined and photographed using a Nikon Eclipse Ni-U microscope (Nikon Instruments, Shanghai, China) in a non-blind manner. For each tissue block, at least 5 non-consecutive sections were studied.

### Immunohistochemistry and immunofluorescence

For immunohistochemistry, tissue sections were deparaffinized, and antigens retrieved by heating the samples in 10 mM sodium citrate buffer (pH 6.0) in a microwave oven for 15 min. Endogenous peroxidases were inactivated by incubation with 3% (v/v) hydrogen peroxide at room temperature for 10 min. Samples were blocked with 1% bovine serum albumin at room temperature for 30 min, and incubated with various primary antibodies at 4°C overnight. After washing, the samples were incubated with biotin-conjugated secondary antibodies at room temperature for 60 min. Slides were developed using Vectastain Elite ABC Kit (from Vector Laboratories, Burlingame, CA, USA) and diaminobenzidine (DAB) substrate. All slides were counterstained with hematoxylin. Negative controls were performed for all experiments by replacing the primary antibody with a non-immune IgG. For semi-quantitative immunoreactivity analysis, at least 5 non-consecutive slides from each sample were processed using Image-J software (NIH, USA) and the data averaged. Image-J was running with Color Segmentation Plugin (for defining positive areas) (http://bigwww.epfl.ch/sage/soft/colorsegmentation/) and IHC Profiler Plugin (for defining positive cells) [56]. For immunofluorescence, cells cultured on glass slides were briefly fixed in 4% paraformaldehyde, permeabilized with 0.1% Triton X-100, and blocked with 1% albumin. Cells were incubated with diluted primary antibodies at 4°C overnight followed by fluorophore-conjugated secondary antibodies at room temperature for 2 hr. Fluorescent images were taken with a confocal microscope (Model LSM710, Zeiss, Jena, Germany) and analyzed using Image-J. For each slide, 3 - 5 random high-power fields were selected for analysis. Immunofluorescence staining in tissue sections was performed using the same protocol as immunohistochemistry except for the secondary antibodies used. Slides were counterstained with DAPI for 1 min. Depending on the expression pattern of the target protein, the fluorescent signals were expressed either as the percentage of cells with positive expression (by image binarization) or as an arbitrary unit of mean fluorescence intensity (by measuring the fluorescence cell by cell).

For appropriate analyses of human AAA sections, we carried out preliminary experiments to examine the distribution of smooth muscle and macrophage cells in the tunica media. We found a very heterogeneous cellular distribution in the aneurysmal wall. Some areas were poorly cellularized and these were excluded from further analysis. Otherwise, SMA generally showed evenly distributed staining; in contrast, macrophages (CD68^+^) showed rather clustered distribution in adjacent to smooth muscle cells. Therefore, for appropriate data analyses we only included the smooth muscle-rich areas with minimal macrophage infiltrations pre-defined using serial sections.

### Cell culture and virus infection

The mouse aortic smooth muscle cell line (MOVAS) was purchased from ATCC (CRL-2797, Manassas, VA) and cultured in Dulbecco's modified Eagle's medium supplemented with 10% fetal bovine serum. Cell cultures were maintained in a humidified atmosphere under 5% CO_2_ at 37^o^C. Twenty-four hours before lentiviral vector infection, cells were subcultured into a 12-well plate (10^5^ cells per well). Cells were incubated with 10^7^ viral particles per well with 5 μg/mL Polybrene in 0.5 mL of complete medium for 8 hr. Then the medium was removed and cells further cultured in normal medium for 3 days before subsequent experiments. Human aortic smooth muscle cells were obtained from ScienCell Research Laboratories (Carlsbad, CA, USA), and maintained in the Smooth Muscle Cell Medium from the same supplier according to instructions.

### Real-time qPCR

Total RNA was extracted using TRIzol Reagent (Thermo Fisher, Waltham, MA, USA). An aliquot of 2 μg of total RNA was reverse transcribed to cDNA using PrimeScript RT Reagent Kit (Takara, Shiga, Japan). Real-time qPCR amplification was performed using Forget-Me-Not EvaGreen qPCR Master Mix (High ROX) (Biotium, Hayward, CA, USA) in a StepOnePlus qPCR system (Thermo Fisher). The primer sequences used in the study were listed in Supplementary Table 1. The qPCR data were analyzed using the 2^-ΔΔCt^ method. The level of 45S pre-rRNA was normalized with the total 18S mature rRNA. For other genes, GAPDH or β-actin was used as the housekeeping gene.

### Proliferation, cell cycle, and senescence-associated β-galactosidase (β-Gal) assays

Cell proliferation was assessed using CCK-8 assay kit (Dojido, Kumamoto, Japan). Briefly, 3 - 5×10^3^ cells in 100 μL of medium were plated into a 96-well plate and cultured for 0 to 72 hr. CCK-8 reagent of 10 μL was added to each well, and further incubated for 2 hr at 37°C. Absorbance at 450 nm was measured using a EMax Plus microplate reader (Molecular Devices, San Jose, CA, USA). Cell cycle was analyzed using a FACS Calibur system (BD Biosciences, San Diego, CA). Cells were detached with 0.25% trypsin, passed through a 70-μm strainer, and fixed overnight in cold ethanol. Propidium iodide staining was performed using a kit from Abcam (ab139418) according to the manufacturer’s instructions. β-Gal staining was performed using Senescence Detection Kit from Abcam (ab65351) according to the manufacturer’s instructions.

### Alkaline comet assay

Alkaline comet assay was performed using Trevigen CometAssay® Kit (Trevigen, Gaithersburg, MD, USA). Detached cells were resuspended in cold PBS to a concentration of 10^5^ cells/mL. An aliquot of 10 μL of cell suspension was added to 100 μL of melted LM agarose maintained at 37°C. The agarose mixture was transferred onto a comet slide and allowed to solidify at 4^o^C. Then the cells were lysed in pre-chilled lysis solution for 60 min at 4°C, and denatured at room temperature in an alkaline solution containing 300 mM NaOH and 1 mM EDTA for 60 min. Electrophoresis was carried out under 1 V/cm condition in a solution (300 mM NaOH and 1 mM EDTA) for 30 min. All incubations and electrophoresis were performed in dark. The slides were stained with SYBR Green for 20 min and immediately photographed using a fluorescence microscope (Nikon Eclipse Ni-U). Comet tail was analyzed using the Comet Assay Software Project tool [57].

### Statistical analysis

Data were analyzed using Prism 7.0 (GraphPad Software Inc, San Diego, CA, USA) and reported as mean ± standard deviation (S.D.). Mean data between two groups were compared by unpaired *t*-test; three or more groups were compared by one-way ANOVA followed by *post hoc* Newman–Keuls test. *P* values < 0.05 (two-tailed) were considered as statistically significant.

## Supplementary Material

Supplementary Figures

Supplementary Tables
