# Supplementary material for: Nucleolar stress induces a senescence-like phenotype in smooth muscle cells and promotes development of vascular degeneration
Source: Aging (Albany NY). 2020 Nov 4;12(21):22174–98. doi: 10.18632/aging.104094 (PMC7695416; doi:10.18632/aging.104094)
Supplement: Supplementary Figures [file aging-12-104094-s001..pdf]

SUPPLEMENTARY FIGURES

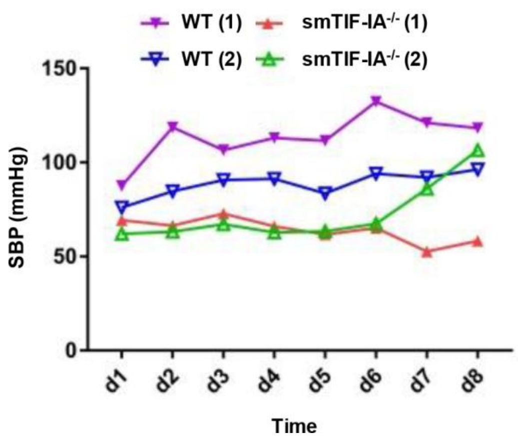

Supplementary Figure 1. Comparison of conscious systolic blood pressures (SBP) in two smTIF-IA$^{-/-}$ mice versus two wild type littermates (WT), which were monitored for an 8-day period using a tail-cuff method (BP-2010A System, Softron Biotechnology, Beijing, China).

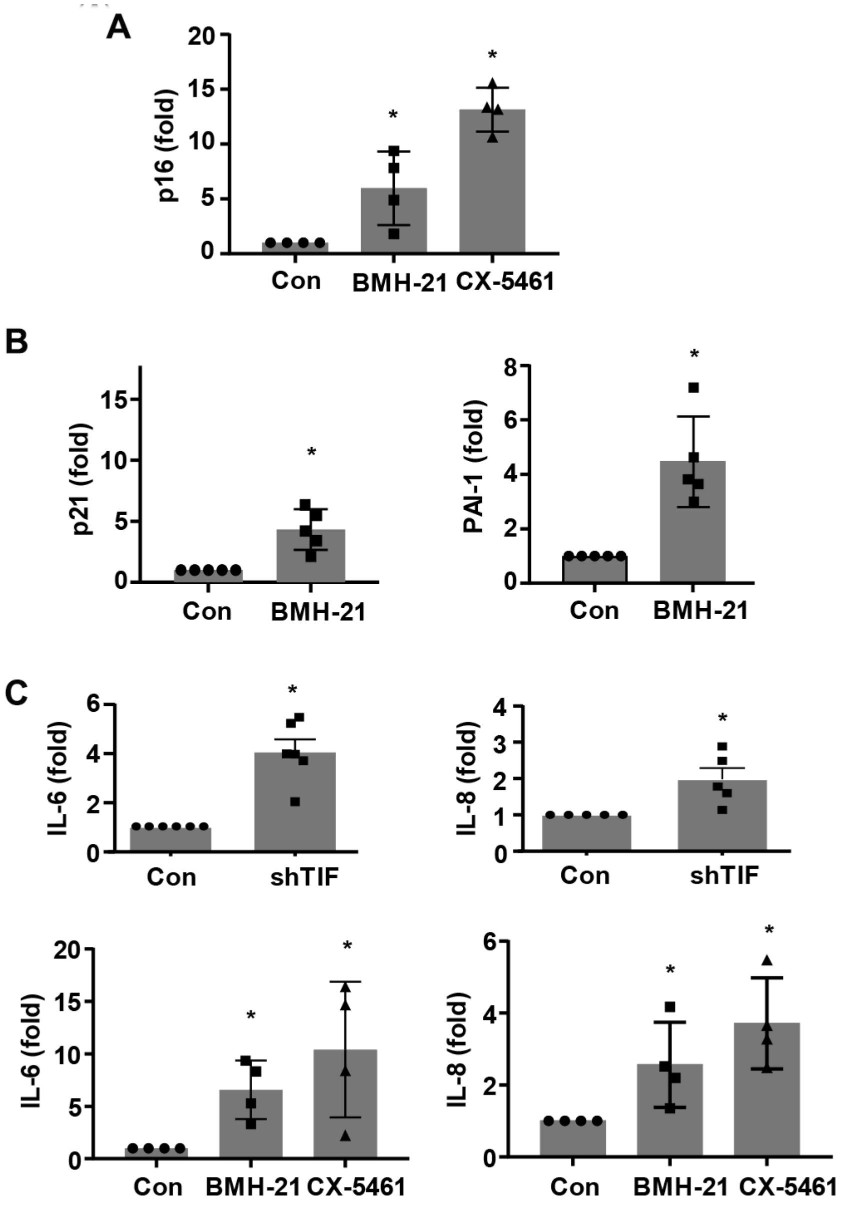

Supplementary Figure 2. Effects of rDNA transcription inhibition on levels of various senescence markers in MOVAS cells detected by real-time PCR. (**A**) Effects of CX-5461 (0.5 μM) and BMH-21 (0.5 μM) on the expression level of $p16^{INK4a}$. (**B**) Effects of BMH-21 (0.5 μM) on the expression levels of $p21^{Cip1}$ and PAI-1. (**C**) Effects of TIF-IA shRNA (shTIF), CX-5461 and BMH-21 on the expression levels of IL-6 and IL-8. * $P < 0.05$ versus control (Con), unpaired *t*-test or one-way ANOVA. The *n* number in each group was represented by the dots.

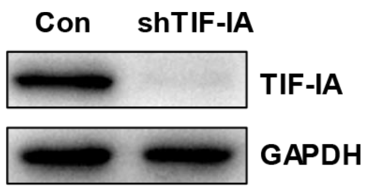

Supplementary Figure 3. Example of western blots showing the effect of lentivirus-mediated TIF-IA shRNA transduction on the protein expression of TIF-IA in MOVAS cells. A non-targeting sequence was used as control (Con).

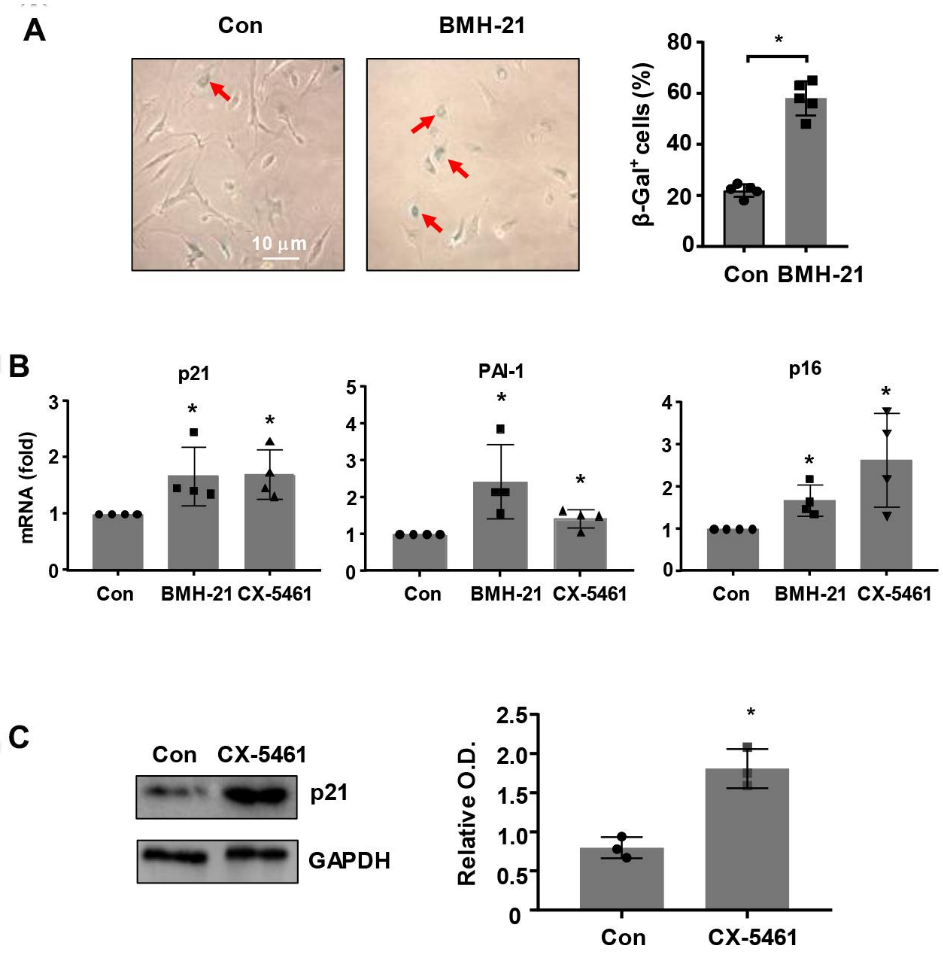

Supplementary Figure 4. Effects of rDNA transcription inhibition on various senescence markers in human aortic smooth muscle cells (HASMCs). (**A**) Effects of BMH-21 (5 μM) on the number of β-Gal-positive cells (blue color, arrows) after 5 days in culture. (**B**) Real-time PCR results showing CX-5461 (0.5 μM) and BMH-21 (5 μM) effects on the expression levels of $p21^{Cip1}$, PAI-1 and $p16^{INK4a}$. (**C**) Representative western blot and mean densitometry data showing the effect of CX-5461 on the protein level of $p21^{Cip1}$. * $P < 0.05$ versus control (Con), unpaired *t*-test or one-way ANOVA. Data were mean ± SD. The *n* number in each group was represented by the dots.

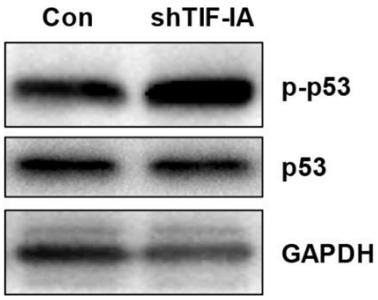

Supplementary Figure 5. Representative western blot images showing increased p53 phosphorylation in TIF-IA gene-silenced MOVAS cells. A non-targeting shRNA sequence was used as control (Con).

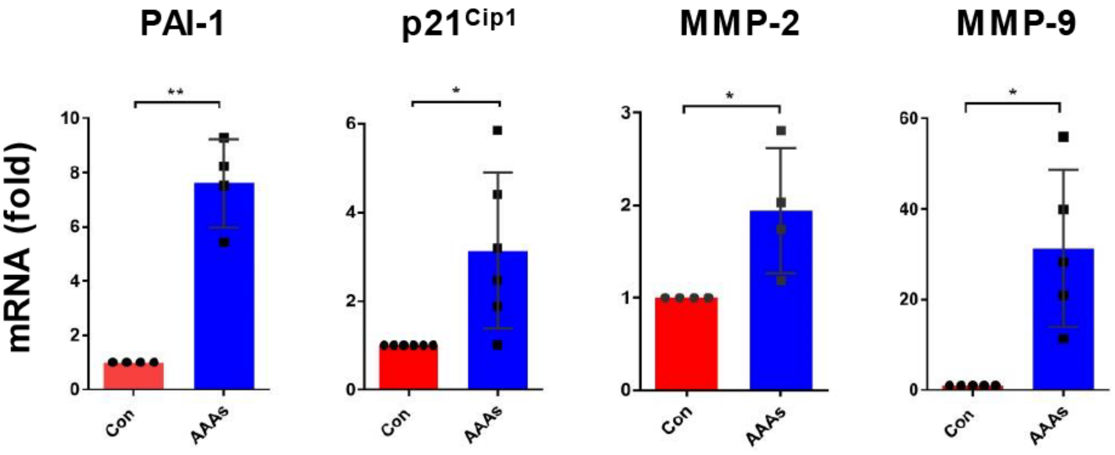

Supplementary Figure 6. Real-time PCR results showing changes in the expression of PAI-1, p21^{Cip1}, MMP-2 and MMP-9 in human AAA tissues. Data are expressed as dot blot-combined bar graphs representing mean ± SD. * $P < 0.05$, ** $P < 0.01$, unpaired *t*-test.

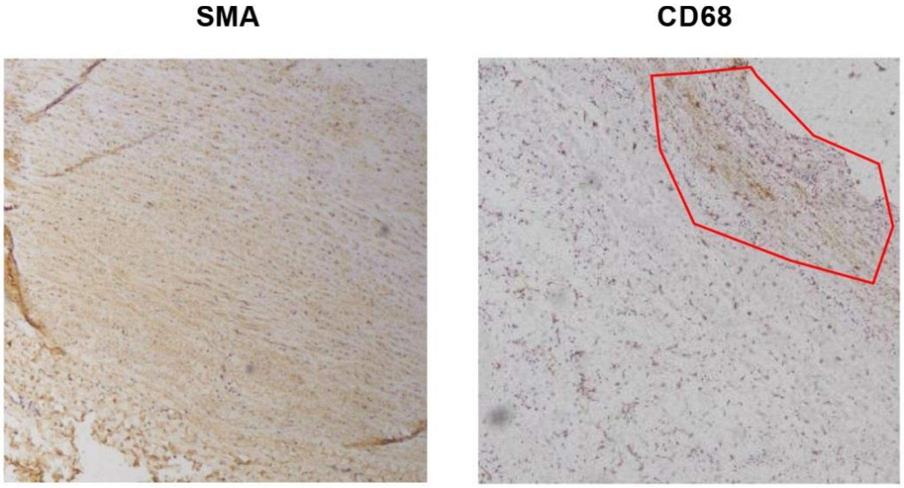

Supplementary Figure 7. Immunohistochemistry images showing different distribution patterns of smooth muscle cells (SMA staining) and macrophages (CD68 staining) in human AAA tissues. Sections were counterstained with hematoxylin. The red line indicated a macrophage-rich area. SMA, smooth muscle α-actin. Low power (40X) fields.

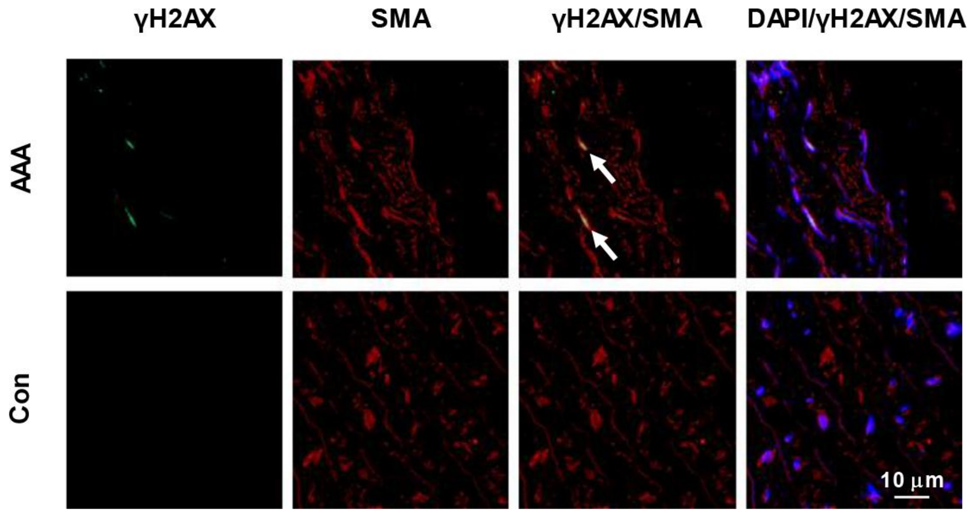

Supplementary Figure 8. Double immunofluorescence labeling images showing that in human AAA sections, the majority of γH2AX signals co-localized with smooth muscle α-actin (SMA). Nuclei were counterstained with DAPI.

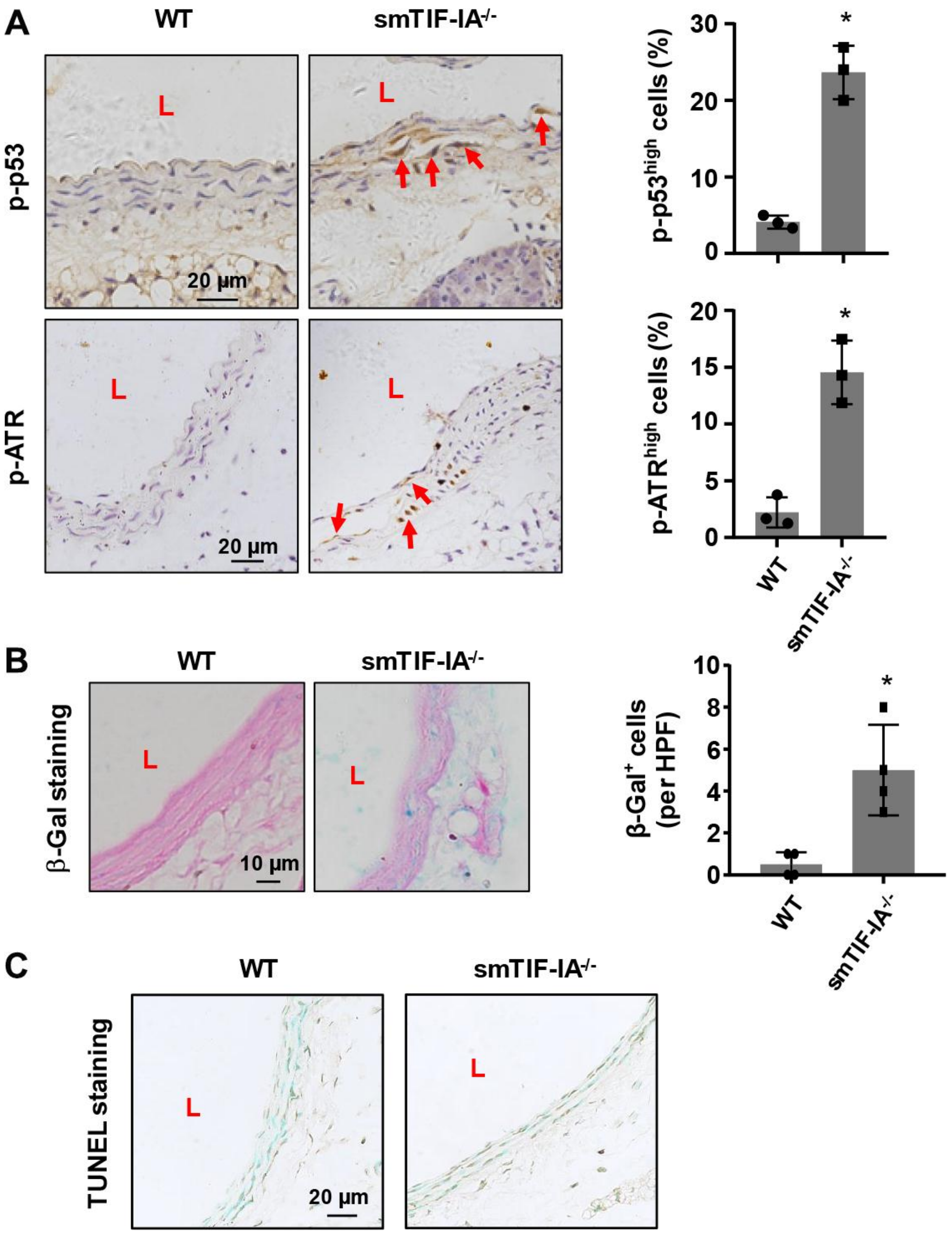

Supplementary Figure 9. Changes in markers of senescence and apoptosis in the tunica media of smTIF-IA$^{-/-}$ aorta. (**A**) Immunohistochemistry data showing the levels of phospho-p53 and phospho-ATR (arrows). Sections were counterstained with hematoxylin. (**B**) Representative images of β-Gal staining and quantitative data showing the presence of senescent cells (blue color) in the tunica media of smTIF-IA$^{-/-}$ aorta. (**C**) Terminal deoxynucleotidyl transferase dUTP Nick-End Labeling (TUNEL) staining showing that smTIF-IA$^{-/-}$ did not induce obvious cell apoptosis in the aorta, comparing with the wild type (WT) control. Data were mean ± SD. * $P < 0.05$ versus WT, unpaired *t*-test. L, lumen side of the vessel. HPF, high power field (100X oil immersion lens).

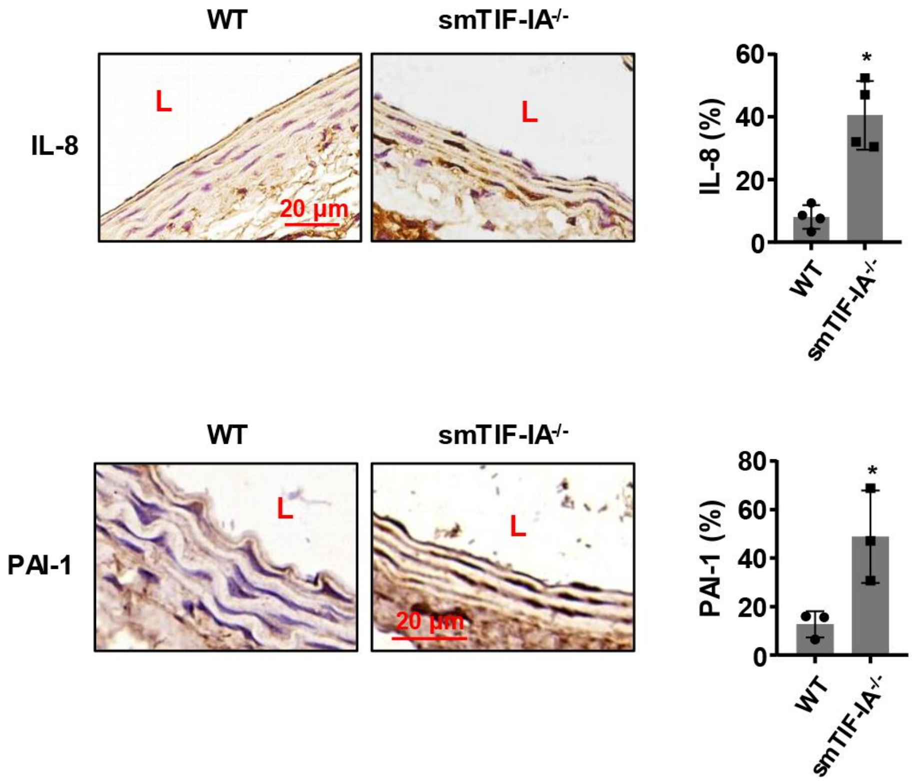

Supplementary Figure 10. Changes in expression of SASP markers IL-8 and PAI-1 in smTIF-IA$^{-/-}$ aorta detected by immunohistochemistry. Sections were counterstained with hematoxylin. Semi-quantitative data of immunoreactivity (% area) are shown on the right. Data were mean ± SD. * $P < 0.05$ versus wild type (WT), unpaired *t*-test. The *n* number in each group was represented by the dots. L, lumen side.
