# Supplementary material for: Nucleolar stress induces a senescence-like phenotype in smooth muscle cells and promotes development of vascular degeneration
Source: Aging (Albany NY). 2020 Nov 4;12(21):22174–98. doi: 10.18632/aging.104094 (PMC7695416; doi:10.18632/aging.104094)
Supplement: Supplementary Tables [file aging-12-104094-s002..pdf]

SUPPLEMENTARY TABLES

Supplementary Table 1. PCR primer sequences used in the study.

TIF-IA (for genotyping)	F: CCGGTGGTCCTGCTTACACTAGAGATGTGG<br>R: AATATAATTTGCAGCAGCCTGCCTGATGATGG
Cre (for genotyping)	F: ACTAAACTGGTCGAGCGATGGA<br>R: TGTCCAGACCAGGCCAGGTA
UBF (Mouse)	F: AGAGAGGCAGTACAAGGTGC<br>R: TGGTCCGGCTAGACTTGGG
TIF-IA (Mouse)	F: AGGATCCAAATAACCCCGCC<br>R: AAGGTACATGTGCAGCCAGT
TBP (Mouse)	F: CCAGACCCCACAACTCTTCC<br>R: CTCAGAAGCTGGTGTGGCAG
RPA43 (Mouse)	F: TAACGCCTCTATCCCTAA<br>R: TTCCAGTTCATCGCCTAC
pre-rRNA (Human)	F: CGATCTGAGAGGCGTGCCTT<br>R: GGCAGCGCTACCATAACGGA
TIF-IA (Human)	F: CCCGGCAGGGTATTGAAGATG<br>R: AACAATCCTTCCGTGGAATCTG
TBP (Human)	F: CAGGGGTTCAGTGAGGTCG<br>R: ACCCTGGGTCACTGCAAAG
UBF (Human)	F: ATGCGAAACTCCACCCTGAG<br>R: TCATGATCTCATCTGGCCGC
Polr1A (Mouse)	F: TGCGTTGCGGGTGATAGA<br>R: TCCCAAAGCGATTGAGTG
PAI-1 (Mouse)	F: CAAGCTCTTCCAGACTATGGTG<br>R: ACCTTTGGTATGCCTTTCCAC
p21 (Mouse)	F: GGGTGAGGAGGAGCATGAAT<br>R: GACAACGGCACACTTTGCTC
18S rRNA (Human)	F: AAACGGCTACCACATCCAAG<br>R: CCTCCAATGGATCCTCGTTA
GAPDH (Mouse)	F: GGACACTGAGCAAGAGAGGC<br>R: TTATGGGGGTCTGGGATGGA
β-actin (Human)	F: TCCAAATATGAGATGCGTTGTTAC<br>R: GTGGACTTGGGAGAGGACTG

Supplementary Table 2. Basic information of the human subjects involved in the study.

	**AAA (n = 6)**	**Donor (n = 6)**
Age	65.5 ± 10.1	42.7 ± 7.5
Male/Female	5/1	5/1
History of hypertension	83.3%	N/A

Age data are mean ± S.D.
